# Application of a haematopoetic progenitor cell-targeted adeno-associated viral (AAV) vector established by selection of an AAV random peptide library on a leukaemia cell line

**DOI:** 10.1186/1479-0556-6-12

**Published:** 2008-09-12

**Authors:** Marius Stiefelhagen, Leopold Sellner, Jürgen A Kleinschmidt, Anna Jauch, Stephanie Laufs, Frederik Wenz, W Jens Zeller, Stefan Fruehauf, Marlon R Veldwijk

**Affiliations:** 1 Department G402, Pharmacology of Cancer Treatment, German Cancer Research Center, INF 280, D-69120, Heidelberg, Germany; 2 Department of Internal Medicine V, University of Heidelberg, INF 410, D-69120, Heidelberg, Germany; 3 Department F010, Applied Tumor Virology, German Cancer Research Center, INF 242, D-69120, Heidelberg, Germany; 4 Institute of Human Genetics Heidelberg, University of Heidelberg, INF 366, D-69120 Heidelberg, Germany; 5 Department G360, Molecular Oncology of Solid Tumors, German Cancer Research Center, INF 580, D-69120, Heidelberg, Germany; 6 Department of Radiation Oncology, Mannheim Medical Center, University of Heidelberg, Theodor-Kutzer-Ufer 1-3, D-68135, Mannheim, Germany; 7 Center for Tumor Diagnostics and Therapy, Paracelsus-Klinik, Am Natruper Holz 69, D-49046, Osnabrück, Germany

## Abstract

**Background:**

For many promising target cells (e.g.: haematopoeitic progenitors), the susceptibility to standard adeno-associated viral (AAV) vectors is low. Advancements in vector development now allows the generation of target cell-selected AAV capsid mutants.

**Methods:**

To determine its suitability, the method was applied on a chronic myelogenous leukaemia (CML) cell line (K562) to obtain a CML-targeted vector and the resulting vectors tested on leukaemia, non-leukaemia, primary human CML and CD34^+ ^peripheral blood progenitor cells (PBPC); standard AAV2 and a random capsid mutant vector served as controls.

**Results:**

Transduction of CML (BV173, EM3, K562 and Lama84) and AML (HL60 and KG1a) cell lines with the capsid mutants resulted in an up to 36-fold increase in CML transduction efficiency (K562: 2-fold, 60% ± 2% green fluorescent protein (GFP)^+ ^cells; BV173: 9-fold, 37% ± 2% GFP^+ ^cells; Lama84: 36-fold, 29% ± 2% GFP^+ ^cells) compared to controls. For AML (KG1a, HL60) and one CML cell line (EM3), no significant transduction (<1% GFP^+ ^cells) was observed for any vector. Although the capsid mutant clone was established on a cell line, proof-of-principle experiments using primary human cells were performed. For CML (3.2-fold, mutant: 1.75% ± 0.45% GFP^+ ^cells, p = 0.03) and PBPC (3.5-fold, mutant: 4.21% ± 3.40% GFP^+ ^cells) a moderate increase in gene transfer of the capsid mutant compared to control vectors was observed.

**Conclusion:**

Using an AAV random peptide library on a CML cell line, we were able to generate a capsid mutant, which transduced CML cell lines and primary human haematopoietic progenitor cells with higher efficiency than standard recombinant AAV vectors.

## Background

Chronic myelogenous leukaemia (CML) represents about 15–20% of all cases of adult leukaemia in Western populations [1]. Currently, the most effective and best tolerated drug against CML is imatinib mesylate (Gleevec^® ^or Glivec^®^, previously known as STI-571), a selective BCR-ABL tyrosine kinase inhibitor [2]. However, resistance of CML cell clones to imatinib mesylate remains a serious clinical challenge [3-5]: over 5% of patients in early stage CML do not achieve complete haematological remission. Of those who do, a constant rate of 4%–6% per year suffer the risk of relapsing [6]. In order to eliminate residual resistant CML cells in patients, novel therapeutic options, for instance gene therapy, have to be considered.

A potential approach would be the transfer of vectors containing either suicide or immune-stimulating genes into these cells. For a gene-based therapy of CML, most standard vector systems lack the required gene transfer efficiency and/or the *in vivo *selectivity. An advancement in vector development for the small parvovirus adeno-associated virus (AAV) by Müller and colleagues (suggested reading [7]) now allows the generation of rAAV capsid mutants that offer higher gene transfer efficiency and a potentially higher target cell specificity. To this end, an AAV random peptide library was used which displays a random seven amino acid peptide sequence within the VP capsid protein domain (at Arg588) that is normally required for binding of AAV2 to one of its natural receptors, heparan sulphate [8]. During the selection of the AAV random peptide library on the target cells, mutants with good binding characteristics to a target receptor are able to transduce the cells, replicate and are propagated during further selection rounds. These mutants may show increased transduction efficiency on and/or increased specificity for the target cells, which has to be assessed in further assays.

Recombinant viral vectors based on AAV exhibit several beneficial features for gene therapy purposes, due to the lack of pathogenicity, high virion stability and its relatively low immunogenicity [9,10]. Although the primarily extra-chromosomal residence of the virus makes them unsuitable for long-term expression in rapidly dividing tissues, it renders it highly favourable for "hit and run" applications (e.g.: drug- or radio-resistance gene transfer and especially gene correction by homologous recombination) in these cell types, without the potential dangers associated with integration (insertional mutagenesis) and long-term exposure to unphysiological transgene levels.

rAAV2 vectors have been used extensively in many clinical and pre-clinical studies, including, for instance the treatment of clotting factor disorders[11], cystic fibrosis [12] and several types of cancer [13-15]. Attempts to efficiently transfer genes into primary human CML cells were prevented by the low susceptibility of the target cells to the vector (unpublished observations). Of note, in general the susceptibility of primary human haematopoietic progenitor cells seems to be highly dependent on both the progenitor source (e.g.: G-CSF-mobilised peripheral blood progenitor cells [16], bone marrow [17], cord blood [18]) and displays a high inter-patient/donor variability [17]. In AAV binding experiments, Ponnazhagan and colleagues [17] showed that the susceptibility or the lack thereof in human haematopoietic progenitors highly correlates with binding of the virus to and subsequent entry into the cell. This suggests that binding of the virus to a suitable receptor on the cell is a rate limiting step [19]. Since high gene transfer efficiency is a prerequisite for any gene therapy approach, methods by Muller and colleagues [7], as well as a similar approach developed by another group [20] may help to overcome this limitation by facilitating binding of AAV capsid mutant to other on primary human haematologic progenitors available receptors and thus allowing entry into these cells. Several groups have previously shown that incorporation of a variety of amino acid sequences into the heparin-binding motif of the AAV2 capsid retargeted the vector to cells previously refractory to the vector [21,22], yet often with low efficiency.

In this investigation, we determined the suitability of an AAV random peptide library on a CML cell line for generating a more efficient and specific rAAV vector for the transduction of leukaemia cell lines and primary cells.

## Methods

### Cells and cell culture

The embryonic kidney cell line 293T and the cervix carcinoma line HeLa-RC [23] were kindly provided by Dr. Kleinschmidt (DKFZ, Heidelberg, Germany) and maintained in Dulbecco's modified Eagle's medium (DMEM, all media and supplements from Gibco Invitrogen Corporation, Karlsruhe, Germany) supplemented with 10% FCS and 5 μg/ml penicillin/streptomycin. All other cell lines were obtained from the tumour cell bank of the German Cancer Research Center (Heidelberg, Germany). The leukaemia cell lines (BV173, EM3, HL60, K562 and Lama84), H-Meso1 and RM-HS-1 cells were cultured in Roswell Park Memorial Institute (RPMI) medium supplemented with 10% FCS and 5 μg/ml penicillin/streptomycin. Primary human CML and peripheral blood progenitor cells (PBPC) were provided by the Department of Internal Medicine V (Heidelberg, Germany): CML cells were isolated from peripheral blood of patients in blast crisis phase, whereas PBPCs were obtained from leukapheresis product of patients with non-myeloid malignancies. Primary human cells were grown in Iscove's Modified Dulbecco's Medium (IMDM), supplemented with 20 ng/ml TPO, 100 ng/ml FL3 and 100 ng/ml SCF (SCF from Genzyme, Cambridge, MA, USA, TPO and FLT-3 from R&D Systems, Minneapolis, MN, USA).

The investigation on viral gene transfer was approved by the Ethical Committee of the Medical Faculty of the University of Heidelberg and informed consent was obtained from each patient.

### Selection and identification of K562-targeted AAV random peptide library clones

For the selection (four rounds), an AAV random peptide library, as described previously by Müller and colleagues [7], was used containing a random 7 amino acid sequence inserted at Arg588 of the Cap gene (position 3967 of AAV2). Within each round, cells were incubated with the AAV random peptide library supernatant for 2 hours, washed and co-infected with adenovirus (kindly provided by the Dr. Balter, vector core of the university hospital of Nantes, Nantes, France). After further incubation for 3 days, cells were harvested and three freeze-thaw cycles were performed to extract the viral particles.

An aliquot of the supernatants of round 2–4 were purified with Qiagen DNA purification kit (Qiagen, Hilden, Germany) and served as a template for a the amplification of the random peptide insert by PCR (Forward primer: 5' CCT GTT ACC GCC AGC AGC GA-3', Reverse primer: 5'-GGT GGC CGC CTG GGC-3').

PCR products were analysed by gel electrophoresis, the PCR band was excised and purified with Qiagen gel purification KIT (Qiagen, Hilden, Germany). The purified product was cloned in the TOPO pCR-4 vector and transformed into OneShot^® ^bacteria according to the manufacturer's instructions (Topo TA Cloning Kit, Invitrogen, Carlsbad, CA, USA). Several colonies of each cloning reaction were screened for insert by direct PCR of bacterial colonies (T3 and T7) and cycle sequencing of these was performed using an ABI Prism Genetic Analyzer 310 (Applied Biosystems, Weiterstadt, Germany) according to the manufacturer's instructions. Sequences were analysed using the Chromas^® ^(Technelysium, Tewantin, Australia) and VectorNTI software (Invitrogen).

### Production, purification and concentration of rAAV particles

For production of the CML-targeted rAAV virus stocks, the following plasmids were used: pMRV-Ef1a-hGFP (rAAV2 vector), several pMT187-xx2 (containing the wtAAV2 genome without ITRs) derivates containing the random peptide inserts and pDGΔVP (an rAAV2 helper plasmid containing all genes required for rAAV2 production, but lacking the Cap gene). Clone identity in the pMT187-xx2 plasmids was confirmed by cycle sequencing.

For production of rAAV2 particles by transient plasmid transfection, 3 × 10^6 ^293T cells/dish were seeded in 10 cm dishes (Becton-Dickinson, Heidelberg, Germany). At 40–70% confluency, cells were transfected with 3.5 μg pMRV-EF1a-hGFP, 7 μg pDGΔVP plasmid and 3.5 μg of the pMT187-xx2 (containing the respective clone) using the Metafectene transfection reagent (Biontex Laboratories, Munich, Germany), according to the manufacturer's conditions.

After 48 h, cells were harvested and subsequently lysed by three cycles of freeze-thawing (-80 to 37°C). Lysates were treated with 50 U/ml endonuclease (Benzonase; Sigma-Aldrich) for 30 min at 37°C and centrifuged twice at 2000 g for 15 min to remove cellular debris.

The clear supernatant was subsequently filtered through a 5 and a 0.8 μm pore size filter (both Millipore, Eschborn, Germany) and was then run over a iodixanol gradient, using the procedure by Zholotukin and colleagues [24]. In brief: the lysate was loaded on top of a 4-layer gradient containing 15, 25, 40 and 60% iodixanol (Optiprep; Axis-shield, Oslo, Norway), and run for 2 hours at 302,000 g in a Beckman Ultracentrifuge (50.2 Ti; Beckman, Munich, Germany). The 40% iodixanol layer containing the rAAV2 particles was recovered using a syringe with needle and diluted in one volume of PBS-MK (PBS supplemented with 1 mM MgCl_2 _and 2.5 mM KCl). The virus stock was aliquotted in 250 μl portions and stored at -80°C until further use.

### Functional and real-time quantitative PCR (RQ-PCR) titration of rAAV2 batches

All rAAV2 stocks were titrated using both a functional and a RQ-PCR titration method, as described previously by Veldwijk et al. [25]. For titration of the AAV2 random peptide library clones during selection, primers and probe were modified to recognise a part of the wild type Rep gene (forward primer: 5'-TTGCAAGACCGGATGTTCAAA-3'; reverse primer: 5'-CTTCCTGCTTGGTGACCTTCC-3' and probe: 5'-ACTCACCCGCCGTCTGGATCATGAC-3'). Functional titration was performed as described previously [25].

### Transduction

One day before transduction, cells were seeded into 24 well plates at a density of 2.5 or 5 × 10^4 ^cells/well in 300 μl of the respective medium. In all experiments cells were transduced with either conventional rAAV2 or the CML-targeted virus particles at a multiplicity of infection (MOI) as described in the results section. Seventy-two hours after infection, cells were harvested and analysed.

### Acquisition and analysis

For acquisition and analysis of the gene transfer data, a FACSCalibur flow cytometer (Becton-Dickinson GmbH, Heidelberg, Germany) equipped with an air-cooled 488 nm Argon laser was used. Data were processed using Cellquest software (Becton-Dickinson). Before acquisition, propidium iodide (10 μg/ml; Sigma-Aldrich, Munich, Germany) was added to samples, to exclude dead cells from analysis. Ten thousand events were acquired and GFP was measured on the Fl1-channel and plotted against side-scatter as described previously [26]. Primary CML and CD34^+ ^PBPC were further stained with anti-hCD45-APC and anti-hCD34-PE labelled antibodies (both from Becton-Dickinson GmbH). GFP and antigen expression was measured against uninfected control cells, thereby correcting auto fluorescence and unspecific fluorescence. Mean fluorescence intensity (MFI) is given in arbitrary fluorescence units (afu). The GFP expression is given in percentages of GFP positive cells (compared to mock control levels).

### Fluoresence in situ hybridisation

Metaphase spreads of primary CML patient cells infected with rAAV2 and CML-targeted rAAV2 vectors were prepared by standard protocols. Two-colour FISH using a commercial probe set for the BCR-ABL translocation (QBiogene, Illkirch, France) was performed according to the manufacturer's instructions. Images of 4 metaphase spreads were acquired using a Leica DM RXA epifluorescence microscope (Leica, Bensheim, Germany) equipped with a Sensys CCD camera (Photometrics, Tucson, AZ, USA) and controlled by the Leica Q-FISH software (Leica Microsystems Imaging Solution, Cambridge, UK). Slides were destained and a ReFISH protocol described by Müller et al. [27] was applied to allow multifluor in situ hybridisation (M-FISH) of combinatorially labelled chromosome painting probes [28]. Images of the same 4 metaphase spreads were evaluated and processed using the Leica MCK software.

#### Statistics

Data are given as mean values ± standard deviation. Significance levels were determined using a two-sided unpaired Student t test.

## Results

### Selection and identification of K562-targeted random peptide library clones

To obtain K562-targeted AAV mutant capsid clones, an AAV random peptide library was applied on the CML cell line K562 as described in the "Material and Methods" section. The selective properties of the random peptide library procedure are based on the premise, that during the selection rounds, more efficient clones subsequently dominate less efficient ones (table 1). Those clones found in the final round with the highest number of copies are therefore regarded as the most efficient capsid mutants obtained on the targeted K562 cell line. Since the inserted amino acid sequences EARVRPP and NSVSLYT prevailed after four selection rounds and together cover most of the amino acid patterns observed, these were chosen. A random computer-generated clone was also constructed, containing the insert sequence SFPFVSS and named "random clone" serving as a negative control in our study.

**Table 1:** Observed amino acid sequences of AAV capsid mutant as observed during four screening rounds on K562 cells.

	**Selection round**		
***Insert sequence***	***2***^*nd*^	***3***^*rd*^	***4***^*th*^

GISPRAG	2	2	→
GVSGRPA	2	2	→
NESRVLS	2	2	2.
KDPVRAP	→	2	3.
ASRPP	5	2	3.
**EARVRPP**	→	→	4.
**NSVSLYT**	→	→	5.
Others	8	7	1.

**Total**	**19**	**17**	**18**

The bolded clones EARVRPP and NSVSLYT were chosen for further investigation. → clone not detected.

### Transduction efficiency of K562-targeted random peptide library clones on leukaemia cell lines

**Figure 1:**
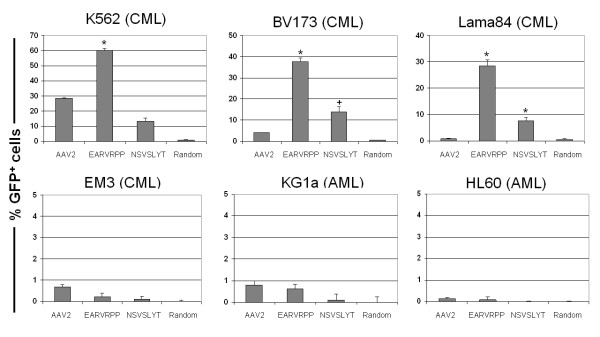
Gene transfer efficiency of the rAAV capsid mutants and control vectors on a panel of leukaemia cell lines (in % GFP^+ ^cells) using an MOI of 100. Cell lines: BV173: CML blast crisis (lymphoid); EM3: CML blast crisis relapse; HL60: AML (M2); K562: CML blast crisis (erythroid); KG1a: AML (erythro-leukaemia); Lama84: CML blast crisis. Random = randomly generated capsid mutant rAAV vector. Data are given as mean ± SD (n ≥ 3). * = rAAV capsid mutants significantly (p < 0.001) more efficient than standard rAAV2. ^+ ^= as *, but with p < 0.01.

To validate the leukaemia targeting efficiency of the selected clones for AAV-mediated gene transfer, four CML (K562, BV173, Lama84, EM3) and two AML cell lines (HL60, KG1a) were transduced with the two AAV library clones (EARVRPP and NSVSLYT), the random clone and a standard rAAV2 vector (Figure 1).

On the targeted cell line K562, a significant increase in gene transfer efficiency of >2-fold (mutant: 60% ± 2% GFP^+ ^cells; p < 0.001) compared to the standard rAAV2 and the random clone could be observed. To an even higher extent (up to 36-fold), this was shown for the CML cell lines BV173 and Lama84 (mutant: 37% ± 2% and 29% ± 2% GFP^+ ^cells, respectively; for both: p < 0.001).

No significant gene transfer (<1% GFP^+ ^cells) into the cell lines EM3 (CML), KG1a and HL60 (both AML) was observed for neither the K562-targeted clones nor control vectors (Figure 1).

Although the K562-targeted capsid mutant clone EARVRPP was more efficient than K562 clone NSVSLYT on any of the cell lines (by at least 2.5-fold), the latter was still significantly more efficient on the CML cell lines BV173 (p < 0.01) and Lama84 (p < 0.001) compared to the control vectors. Due to the superiority of the capsid mutant clone EARVRPP during the leukaemia cell line screenings and no detectable gene transfer by the K562-targeted clone NSVSLYT in preliminary screenings of primary haematopietic cells, the latter was omitted from further experiments.

For the random capsid mutant clone, in none of the leukaemia cell lines significant gene transfer was observed (<1% GFP^+ ^cells). Similar results were obtained using primary blood progenitors (data not shown).

### Determining the specificity of the K562-targeted clones on solid tumour cell lines

Although a significant increase in gene transfer efficiency was found with the K562-targeted clone EARVRPP in three of the leukaemia cell lines, no complete CML-specificity could be observed in those experiments. In order to further investigate the specificity of the targeted vectors, a panel of three generally rAAV2-susceptible solid tumour cell lines (HeLa-RC, H-Meso1 and RM-HS-1) [13,16,29] were transduced with the K562-targeted and control vectors, and gene transfer efficiency (% GFP^+ ^cells) as well as expression level (MFI; mean fluorescence intensity) determined. Standard rAAV2 vectors were significant (for all three lines: p < 0.01) more efficient than the rAAV capsid mutants, although all three non-leukaemia cell lines could readily be transduced with any of the vectors (table 2). Since on all cell lines a reduction in both %GFP^+ ^cells and MFI for the K562-targeted clone EARVRPP compared to the standard rAAV2-treated cells could be observed, an increase in leukaemia cell and a reduction in non-leukaemia cell specificity of the clone can be suggested.

**Table 2:** Gene transfer efficiency of K562-targeted and standard rAAV2 vectors on solid tumour cells.

	**%GFP^+ ^cells**		**MFI (AU)**	
**Cell line**	**EARVRPP**	**rAAV2**	**EARVRPP**	**rAAV2**

RM-HS-1	46.01 ± 10.82	97.61 ± 3.21	35.03 ± 12.93	395.64 ± 52.93
H-Meso1	76.42 ± 2.41	88.30 ± 4.34	70.29 ± 9.54	150.89 ± 23.22
HeLa-RC	77.94 ± 6.60	98.22 ± 1.21	32.57 ± 7.73	143.49 ± 12.21

A significant (p < 0.05; irrespective of cell line) superiority of the standard rAAV2 vector on three solid tumour cell lines could be shown for both %GFP^+ ^cells and MFI. MFI: mean fluorescence intensity; AU: Arbitrary units.

### Transduction of primary peripheral blood progenitor cells (PBPC) and CML cells

**Figure 2:**
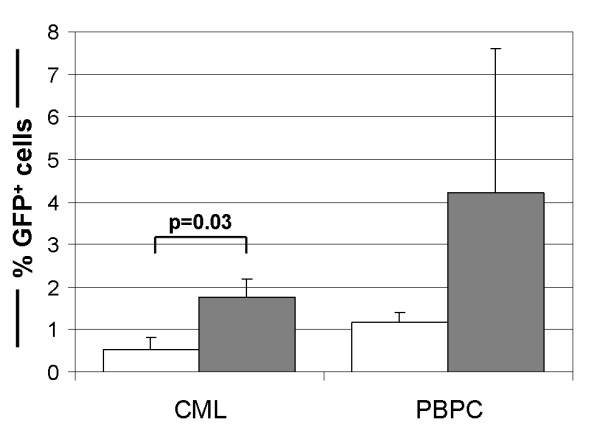
Gene transfer efficiency of the rAAV capsid mutant (EARVRPP; ) and a standard rAAV2-based vector (□) on primary human CML and CD34^+ ^PBPC (in % GFP^+ ^cells) using an MOI of 100. Data are given as mean ± SD (n ≥ 4).

After the promising results on a panel of leukaemia cell lines showing the increase in gene transfer efficiency and an increased specificity for the K562-targeted vector on leukaemia cells, although not generated on primary human progenitor cells, its efficiency on PBPCs (both leukaemia and normal) was determined in proof-of-principle experiments. Therefore, peripheral blood from CML patients and CD34-selected leukapheresis product from patients with non-myeloid diseases were transduced (both n ≥ 4) with either the K562 clone EARVRPP or a standard rAAV2 vector. Mock transduced sample served as control. Although the AAV capsid mutant was established onto a CML cell line, for both CML cells (%GFP^+^_aav2_: 0.54% ± 0.29%; %GFP^+^_mut_.: 1.75% ± 0.45%) and CD34^+ ^PBPC (%GFP^+^_aav2_: 1.19% ± 0.23%; %GFP^+^_mut_.: 4.21% ± 3.40%) an increase in gene transfer efficiency with the K562 clone EARVRPP was observed compared to the standard rAAV2 vector (Figure 2), which was significant for the CML (p = 0.03) group. The identity of the transduced CML cells was confirmed by multicolor FISH for the BCR-ABL mutation of the CML cells (100%, data not shown). For the normal human primary PBPC, an anti-CD34 co-staining was performed to identify the CD34^+^/GFP^+ ^population.

## Discussion

In this study, a peripheral blood progenitor cell-targeted rAAV2 vector was generated using an AAV random peptide library on a leukemia cell line (K562), which was able to transduce several leukaemia cell lines and primary human blood cells with a significant higher efficiency than standard rAAV2 vectors.

Somatic gene transfer in human blood stem cells has been investigated for over two decades, with mouse onco-retroviral vectors being the most efficient, followed by lentiviral vectors more recently [30,31]. However, the subsequent insertional mutagenesis-induced development of leukaemia in patients treated with retroviral vectors for severe combined immunodeficiency, although successfully cured for the latter condition, put a spotlight on the risk of those vectors [32]. The safety profile of lentiviral vectors under clinical settings is still unclear. Of note: although theoretically rAAV-based vectors could induce insertional mutagenesis, the probability is very low due to their episomal residence.

Thus AAV-based vectors, which have not yet been associated with any disease, offer a promising alternative. Although rAAV2 vectors have been used for efficient transduction in various cells and tissue types, and are used in clinical trials [33-36], the transduction of primary human CML cells or CD34^+ ^PBPC had been hindered by the low susceptibility of those cells. Some investigators had initially concluded that human haematopoietic stem cells could not be transduced at all [16,26,37], whereas others mentioned high vector-to-cell ratios as a prerequisite of high gene transfer rates [38,39]. Data from several publications that could show detectable gene transfer into blood stem cells suggest that the source (e.g: cord blood, bone marrow, G-CSF-mobilised PBPC; human vs mouse) of the cells seem to be of high relevance [18,40]. However, most of the obstacles to AAV-mediated haematopoietic stem cell gene transfer were elucidated by greater insight into the life cycle of AAV, with AAV binding, entry [8,41,42], intra cellular trafficking [43] and second-strand DNA synthesis [44] as key issues.

The goal of our study was to create an AAV-based vector that can efficiently and selectively transduce haematopoietic progenitor cells for further gene therapeutic application (e.g.: radio-protection of PBPC [14] and suicide gene transfer for the treatment of CML [29]). With the AAV random peptide library, we address the AAV entry and binding mechanism, as we manipulate the capsid region known for binding to the cell surface heparane sulfate proteoglycan.

In order to test the suitability of the AAV random peptide library for obtaining a more efficient and selective blood progenitor cell-targeted rAAV vector, the CML cell line K562 was chosen. The first step in our approach was to select and identify K562-targeted clones. Although several clones were successfully isolated during selection, the yield of mutant inserts was hampered by wild type-like AAV "insertless" clones (first generation AAV random peptide library), which might explain why during selection some clones were only observed in the last round without prior appearance.

Using the rAAV capsid mutant clones on a panel of leukaemia cell lines, an over 2-fold increase in gene transfer over standard rAAV2-based vectors could be obtained on the initially targeted K562 cells (Figure 1). On BV173 (9-fold) and Lama84 (36-fold) this ratio was even higher. Only the CML cell line EM3 seemed to be completely refractory (<1%) to gene transfer with any of the vectors. On both AML cell lines (KG1a and HL60), no improvement in gene transfer compared to standard rAAV2 vectors could be observed. These results not only imply a difference between the capsid mutants and the control vectors on a genomic and phenotypic level, but also on a functional level. The receptor expression of the target cells is of high relevance. Apart from known AAV2 receptors, like HSPG [8], the human fibroblast growth factor receptor 1, αvβ5 integrin [41,42] or hepatocyte growth factor receptor, c-Met [45], novel binding moieties may be generated on the vector capsid after its modification using the AAV random peptide library.

The AAV capsid mutants showed pronounced specificity for the screened target cells (a CML cell line); only one CML line (EM3) showed no increase in gene transfer efficiency of the capsid mutants compared to standard rAAV2 vectors. On a panel of non-haematopoietic cell lines, on the other hand, all lines tested showed significantly reduced gene transfer efficiency of the rAAV capsid mutants compared to the standard rAAV2 vectors suggesting altogether an increase in specificity towards haematopoietic versus non-haematopoietic cells.

After the tumour cell lines, the vectors were tested – as a proof-of-principle – on primary human CML and primary CD34^+ ^PBPC. On these, higher gene transfer rates of the rAAV capsid mutants than conventional rAAV2 vectors were observed, which is of note, since the capsid mutants were generated on a cell line. Applying the AAV random peptide library on the primary cells, a potentially higher gene transfer efficiency into primary cells would be expected from the thereby obtained rAAV capsid mutants. A further interesting observation was the high inter-patient variability observed with the capsid mutants on CD34^+ ^PBPC, which has been previously observed by Ponnazhagan and colleagues [17] using standard rAAV2 vectors on primary human bone marrow-derived blood progenitors.

## Conclusion

In summary, an AAV capsid mutant clone could be established on a CML cell line, which was more efficient on both leukaemia cell lines and primary human haematopoietic progenitors than standard rAAV2-based vectors. Although our results on primary human blood progenitor cells do not warrant clinically relevant gene transfer levels, the increase in gene transfer efficiency in both human leukemia cell lines and primary progenitors show that the AAV random peptide library holds promise for the generation of more efficient and selective rAAV-based vectors.

## Declaration of competing interests

The authors declare that they have no competing interests.

## Authors' contributions

Contribution: MS performed, helped with the design of the experiments and prepared the draft manuscript, LS performed some of the experiments and commented on the manuscript; JAK provided advice on the design of the study, provided vital reagents and commented on the manuscript; AJ designed and performed the FISH experiments; SL, FW, SF and WJZ provided advice on the design of the study and commented on the manuscript; MRV conceived, designed and supervised the study, participated in the preparation of and commented on the manuscript.
